# From autophagy–lysosomal deficits to neurodegeneration in Niemann-Pick type C1 disease: implications for age-related neurodegenerative disorders

**DOI:** 10.3389/fnins.2026.1857866

**Published:** 2026-05-18

**Authors:** Yuki Kawachi, Gamze Kocak, Viktor I. Korolchuk, Tetsushi Kataura, Sovan Sarkar

**Affiliations:** 1 Department of Neurology, Institute of Medicine, University of Tsukuba, Tsukuba, Japan; 2 Department of Cancer and Genomic Sciences, School of Medical Sciences, College of Medicine and Health, University of Birmingham, Edgbaston, Birmingham, United Kingdom; 3 Faculty of Medical Sciences, Biosciences Institute, Newcastle University, Newcastle upon Tyne, United Kingdom

**Keywords:** autophagy, cell death, lysosome, mitochondria, NAD, neurodegeneration, neuroinflammation, NPC1

## Abstract

Niemann-Pick type C1 (NPC1) disease is a neurodegenerative lysosomal storage disorder caused by loss-of-function mutations in the *NPC1* gene. NPC1 deficit primarily disrupts lipid homeostasis and subsequently drives cellular degeneration through mechanisms involving impaired autophagy and mitophagy, mitochondrial dysfunction, and, recently demonstrated NAD depletion that links autophagy impairment to neuronal death. Emerging evidence also highlights the activation of innate immune signaling leading to neuroinflammation. In this review, we synthesize current mechanistic insights and describe how these molecular deficits are interconnected to drive neuronal death in NPC1 disease. We also discuss how these pathological processes parallel those observed in major age-related neurodegenerative pathologies such as Alzheimer’s and Parkinson’s disease. Finally, we highlight emerging therapeutic strategies that can potentially ameliorate these cellular deficits, offering avenues for mitigating neurodegeneration in NPC1 disease and other related neurodegenerative disorders.

## Introduction

Niemann-Pick disease type C1 (NPC1) disease is a rare (incidence ~1:100,000), autosomal recessive lysosomal storage disorder (LSD) with a wide spectrum of clinical manifestations, most typically progressive neurodegeneration and hepatosplenomegaly (Vanier, 2010). NPC1 is caused by biallelic pathogenic variants in the *NPC1* gene encoding the NPC1 protein, a cholesterol transporter located on the late endosomal/lysosomal membrane (Li et al., 2017). NPC1 plays a central role in cholesterol egress from endolysosomes, and thereby regulates cholesterol distribution to multiple cellular compartments, including the plasma membrane, endoplasmic reticulum (ER), and mitochondria (Hoglinger et al., 2019). Loss of NPC1 function causes the accumulation of cholesterol as well as sphingolipids within lysosomes (Newton et al., 2018), leading to the disruption of lysosomal homeostasis and subsequent multiple cellular dysfunctions, which collectively contribute to neuronal cell death (Kocak et al., 2025).

Macroautophagy (hereafter referred to as autophagy) is an intracellular degradation pathway in which cytoplasmic components are sequestered within double-membrane structures termed autophagosomes and subsequently degraded following fusion with lysosomes. This process encompasses multiple protein complexes that govern sequential steps including initiation, membrane nucleation, autophagosome formation and maturation, and fusion with lysosomes. Autophagy plays a critical role in maintaining cellular homeostasis by eliminating unwanted macromolecules like abnormal protein aggregates and dysfunctional organelles such as damaged mitochondria (Lahiri et al., 2019). Genetic disruption of essential autophagy genes in normal mice causes neurodegeneration (Hara et al., 2006; Komatsu et al., 2006), and numerous pathological variants in autophagy-related genes have been identified in familial neurodegenerative disorders—highlighting the physiological importance of autophagy in neuronal survival (Klionsky et al., 2021). Indeed, defective autophagy has been reported in several neurodegenerative conditions (Menzies et al., 2017; Seranova et al., 2020). Additionally, lysosomal dysfunction primarily affects the late steps of autophagy involving autophagosome–lysosome fusion and cargo degradation, thereby impairing autophagic flux as observed in certain LSDs and neurodegenerative diseases (Seranova et al., 2017; Nixon and Rubinsztein, 2024).

In this review, we focus on the molecular mechanisms underlying cellular deficits, namely impaired autophagy, mitochondrial dysfunction, inflammatory responses, and recently identified NAD depletion, in NPC1 disease. We further discuss how these processes collectively drive neurodegeneration. Finally, from a “rare-to-common” perspective, we review the shared pathogenic mechanisms between NPC1 disease and major neurodegenerative disorders.

## Autophagy dysfunction in NPC1 disease

Multiple studies have demonstrated an impairment in autophagy associated with buildup of autophagosomes in NPC1 disease. NPC1-deficient cells exhibited aberrant accumulation of autophagosomes and autophagic cargo (Sarkar et al., 2013; Maetzel et al., 2014; Kataura et al., 2024), indicating perturbation of autophagosome–lysosome fusion and defective autophagic flux. The membrane fusion between autophagosomes and lysosomes is mediated by a specific SNARE machinery involving the autophagosomal SNARE syntaxin 17 (STX17) and the endosomal/lysosomal SNARE vesicle-associated membrane protein 8 (VAMP8), along with synaptosomal-associated protein 29 (SNAP-29) (Itakura et al., 2012). In NPC1-deficient cells, this process is disrupted through impaired interaction between STX17 and VAMP8 (Sarkar et al., 2013), leading to defective fusion events. Additionally, NPC1 deficiency leads to decreased expression of VEGF, resulting in reduced activity of sphingosine kinase, the enzyme responsible for sphingosine metabolism (Lee et al., 2014). This results in the accumulation of sphingosine in lysosomes, which disrupts lysosomal calcium (Ca^2+^) homeostasis. Lysosomal Ca^2+^ release serves as a critical signal to facilitate autophagosome–lysosome fusion (Lorincz and Juhasz, 2020), and thus its perturbation affects the vesicle fusion event in NPC1-deficient cells (Lee et al., 2014). Consistent with the phenotype of autophagosome accumulation in NPC1 disease, previous studies have also reported similar observations (Pacheco et al., 2007; Ordonez et al., 2012).

**Figure 1:**
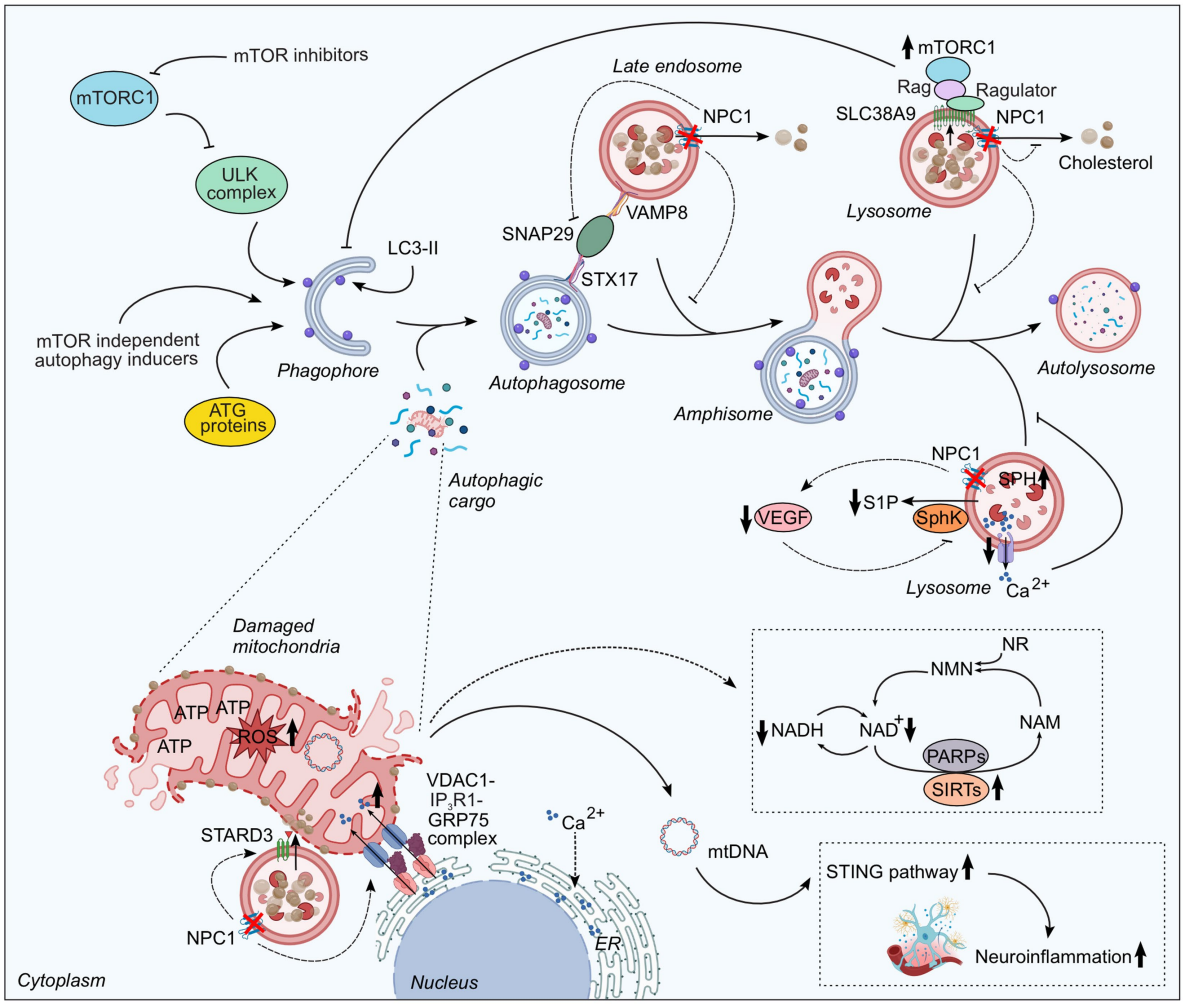
Molecular pathways linking autophagy–lysosomal impairment to neurodegeneration in Niemann-Pick type C1 (NPC1) disease. Schematic overview of the molecular mechanisms underlying autophagy–lysosomal dysfunction in NPC1 disease. Autophagy is initiated through both mTORC1-dependent and mTORC1-independent pathways; inhibition of mTORC1 triggers autophagy through activation of the ULK complex, while several autophagy-related (ATG) proteins mediate the initiation and expansion of autophagic membranes. Loss-of-function mutations in NPC1, which prevent cholesterol efflux leading to its accumulation within late endosomal and lysosomal compartments, impair autophagic flux. Defective autophagy arises due to failure in SNARE machinery involving STX17, VAMP8, and SNAP-29, thereby preventing autophagosome maturation. NPC1 deficiency also reduces vascular VEGF expression, leading to decreased activity of sphingosine kinase (SphK) and consequent accumulation of sphingosine (SPH) within lysosomes that reduces lysosomal calcium (Ca^2+^) release and disrupts autophagosome-lysosome fusion. Accumulation of lysosomal cholesterol further drives mTORC1 hyperactivation via SLC38A9, resulting in suppression of autophagy and mitophagy. In parallel, the buildup of cholesterol and other lipids compromises membrane integrity and proteolytic capacity, directly contributing to impaired autophagic degradation. Moreover, NPC1 deficiency leads to accumulation of damaged mitochondria, characterized by morphological abnormalities, increased ROS production, loss of mitochondrial membrane potential, and impaired respiratory capacity. Altered intracellular cholesterol trafficking promotes aberrant mitochondrial cholesterol accumulation mediated by STARD3, a sterol-binding protein that facilitates cholesterol transfer from lysosomes to mitochondria. In addition, disrupted cholesterol homeostasis remodels ion channel distribution at the plasma membrane, promoting excessive Ca^2+^ transfer from ER to mitochondria via IP_3_R1-GRP75-VDAC1 complex that exacerbates mitochondrial dysfunction and cellular stress. Mitochondrial dysfunction and elevated ROS production contribute to hyperactivation of PARPs and SIRTs, leading to depletion of cellular NAD pool and disruption of the autophagy–NAD axis, which contributes to neurodegeneration. Furthermore, mitochondrial damage results in the release of mtDNA, which activates the STING pathway and triggers inflammatory responses, thereby contributing to neuroinflammation and progressive neurodegeneration. ER, endoplasmic reticulum; GRP75, glucose-regulated protein 75; IP_3_R1, inositol 1,4,5-trisphosphate receptor type 1; mtDNA, mitochondrial DNA; mTORC1, mechanistic target of rapamycin complex 1; NAD, nicotinamide adenine dinucleotide; PARPs, poly(ADP-ribose) polymerases; ROS, reactive oxygen species; S1P, sphingosine-1-phosphate; SIRTs, sirtuins; SNAP-29, synaptosomal-associated protein 29; SNARE, soluble N-ethylmaleimide-sensitive factor activating protein receptor; STARD3, StAR-related lipid transfer domain-containing 3; STING, stimulator of interferon genes; STX17, syntaxin 17; VAMP8, vesicle-associated membrane protein 8; ULK, Unc-51-like kinase; VDAC1, voltage-dependent anion channel 1; VEGF, vascular endothelial growth factor.

Autophagy dysregulation also occurs at the upstream initiation step. The mechanistic target of rapamycin complex I (mTORC1), a multiprotein complex composed of protein kinase mTOR and several regulatory proteins, negatively regulates both the autophagy initiation and the transcription of autophagy/lysosomal genes (Goul et al., 2023). The activation of mTORC1 on the lysosomal membrane occurs through its interactions with the Rag/Ragulator and Rheb/TSC complexes (Cui et al., 2025). Recent studies revealed that lysosomal cholesterol activates mTORC1 via the lysosomal amino acid transporter SLC38A9 (Castellano et al., 2017), and thus excessive lysosomal cholesterol accumulation resulting from NPC1 deficiency leads to hyperactivation of mTORC1 and subsequent suppression of autophagy and mitophagy (autophagic clearance of mitochondria) (Davis et al., 2021). In addition to the perturbations in autophagy initiation and autophagosome–lysosome fusion, aberrant lysosomal storage of cholesterol and other lipids disrupt lysosomal membrane integrity and proteolytic capacity, which may directly contribute to autophagy dysfunction (Ebner et al., 2025) (Figure 1).

Of therapeutic relevance, pharmacological stimulation of autophagy with rapamycin (mTORC1 inhibitor) or with mTORC1-independent autophagy inducers such as carbamazepine, trehalose, BRD5631, celecoxib and memantine restores functional autophagic flux and improves cell viability in NPC1 cell models including disease-affected neurons generated from NPC1 patient-derived induced pluripotent stem cells (iPSCs) (Sarkar et al., 2013; Maetzel et al., 2014; Kuo et al., 2015; Kataura et al., 2024). Notably, carbamazepine, celecoxib and memantine are FDA-approved drugs. Since these drugs induce mTORC1-independent autophagy, which is desirable due to lesser side-effects than the mTORC1 inhibition route (Sarkar, 2013), they represent promising candidates for further clinical evaluation in NPC1 disease.

## Mitochondrial dysfunction in NPC1 disease

Impairment in autophagy can affect mitochondrial function and turnover. Mitochondria are double-membraned organelles that function as central hubs of cellular metabolism and signaling, generating the most ATP in cells through oxidative phosphorylation whilst orchestrating diverse processes including reactive oxygen species (ROS) production, redox signaling and cell death. Mitochondrial dysfunction has long been strongly implicated in the pathogenesis of neurodegenerative disorders including NPC1 disease (Wheeler and Sillence, 2020; Suomalainen and Nunnari, 2024).

In NPC1 disease models, morphological alterations of mitochondria have been observed together with increased ROS production, loss of mitochondrial membrane potential, and impaired respiratory capacity (Yu et al., 2005; Ordonez et al., 2012). Mechanistically, increased mitochondrial cholesterol, elevated mitochondrial Ca^2+^ influx, and impaired mitophagy have been demonstrated in NPC1-deficient cells, which collectively lead to the impairment of mitochondrial function as discussed below.

Although the cholesterol content of mitochondrial membranes is much lower than that of the plasma membrane, tight regulation of mitochondrial cholesterol is essential for maintaining mitochondrial structure and function (Elustondo et al., 2017). In NPC1 disease, mitochondrial cholesterol levels are abnormally increased. This is mediated by sterol-binding protein StAR-related lipid transfer domain-containing 3 (STARD3), which promotes the formation of membrane contact sites between lysosomes and mitochondria, thereby facilitating cholesterol transfer from the lysosomes to mitochondria (Charman et al., 2010; Hoglinger et al., 2019).

In neurons, NPC1 deficiency leads to remodeling of ion channel distribution at the plasma membrane, likely due to altered cholesterol distribution (Levitan et al., 2010). Specifically, clustering of the voltage-gated potassium channel Kv2.1 promotes clustering of the L-type voltage-gated calcium channel CaV1.2, resulting in excessive Ca^2+^ influx into the cytoplasm. The elevated cytosolic Ca^2+^ is subsequently transferred from the ER to mitochondria through the inositol 1,4,5-trisphosphate receptor type 1 (IP_3_R1)—glucose-regulated protein 75 (GRP75)—voltage-dependent anion channel 1 (VDAC1) complex, leading to mitochondrial dysfunction and neuronal cell death via apoptosis and necrosis (Casas et al., 2023).

Mitophagy represents a selective form of autophagy that removes aged, damaged, or excess mitochondria to maintain mitochondrial quality and quantity (Ratliffe et al., 2023; Uoselis et al., 2023). In NPC1-deficient cells, autophagy dysfunction results in reduced mitophagy flux (Davis et al., 2021; Kataura et al., 2024). Pharmacological activation of mitophagy restores mitochondrial membrane potential, suggesting that impaired mitophagy contributes to mitochondrial dysfunction in NPC1 disease (Kataura et al., 2024).

## NAD depletion in NPC1 disease

Autophagy ensures cell survival by maintaining intracellular levels of nicotinamide adenine dinucleotide (NAD) (Wilson et al., 2023). NAD is an essential cofactor involved in redox reactions in mitochondrial respiration and cellular signaling mediated by NAD^+^-dependent enzymes such as sirtuins (SIRTs) and poly(ADP-ribose) polymerases (PARPs) (Covarrubias et al., 2021; Yusri et al., 2025). These enzymes participate in cellular stress responses, particularly DNA repair, and regulation of mitochondrial homeostasis (Canto et al., 2013). Disruption of NAD metabolism has long been implicated in aging and neurodegenerative diseases (Wilson et al., 2023; Kocak et al., 2025; Vinten et al., 2025; Zhang J. et al., 2025).

**Figure 2:**
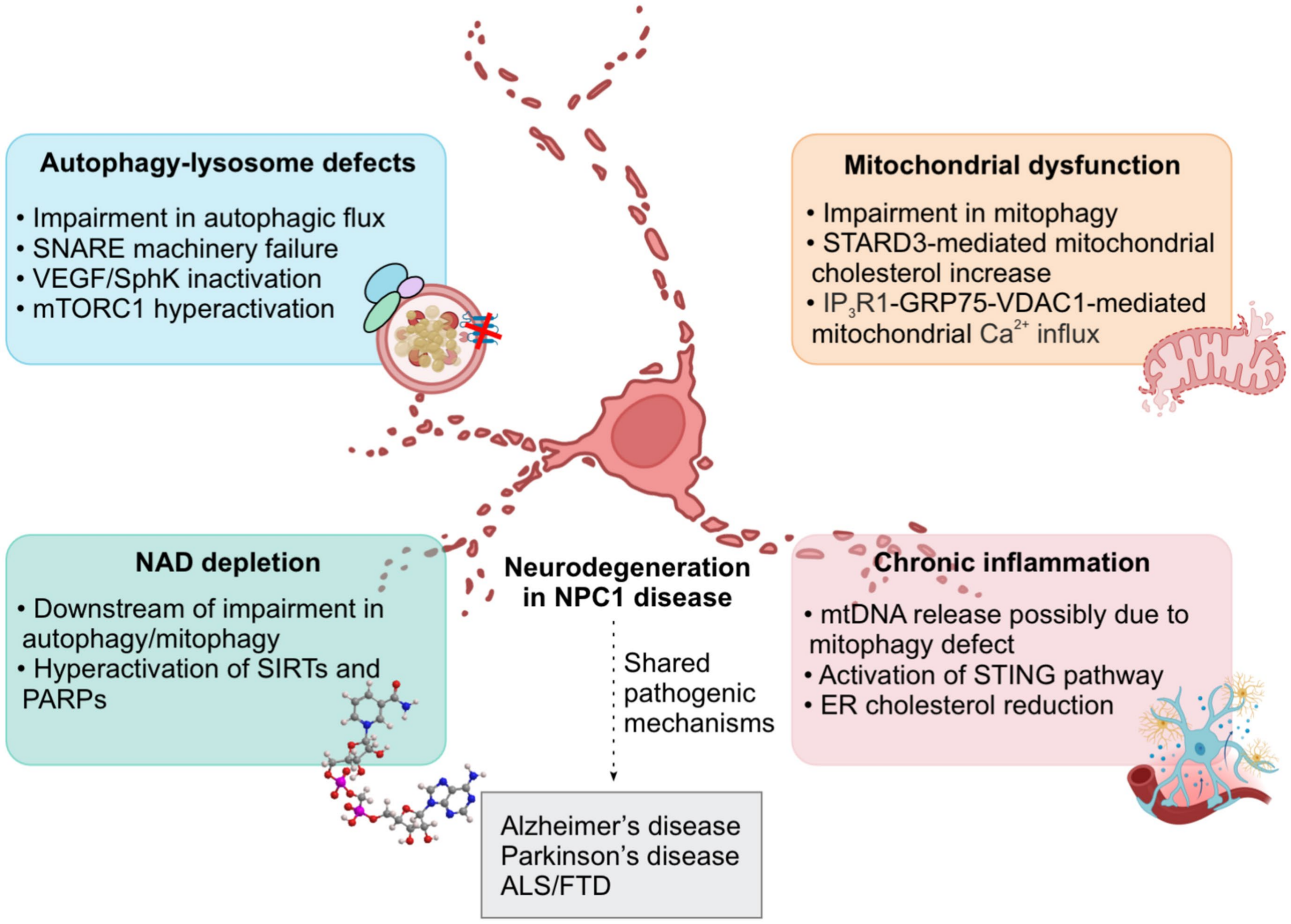
Shared pathogenic mechanisms between Niemann-Pick type C1 (NPC1) disease and other neurodegenerative diseases. Schematic illustration of cellular defects in NPC1 disease, such as: (i) autophagy-lysosome dysfunction characterized by impairment in autophagic flux due to SNARE machinery failure, VEGF/SphK inactivation, and mTORC1 hyperactivation; (ii) mitochondrial dysfunction involving impairment in mitophagy, STARD3-mediated mitochondrial cholesterol increase, and IP_3_R1-GRP75-VDAC1-mediated mitochondrial Ca^2+^ influx; (iii) NAD depletion downstream of impairment in autophagy/mitophagy and due to hyperactivation of SIRTs and PARPs; (iv) chronic inflammation marked by mitochondrial DNA (mtDNA) release possibly due to mitophagy defect, activation of STING pathway, and ER cholesterol reduction. These phenotypes are also common to other neurodegenerative conditions including Alzheimer’s disease, Parkinson’s disease, amyotrophic lateral sclerosis (ALS), and frontotemporal dementia (FTD). GRP75, glucose-regulated protein 75; IP_3_R1, inositol 1,4,5-trisphosphate receptor type 1; mTORC1, mechanistic target of rapamycin complex 1; NAD, nicotinamide adenine dinucleotide; PARPs, poly(ADP-ribose) polymerases; SIRTs, sirtuins; SNARE, soluble N-ethylmaleimide-sensitive factor activating protein receptor; SphK, sphingosine kinase; STARD3, StAR-related lipid transfer domain-containing 3; STING, stimulator of interferon genes; VDAC1, voltage-dependent anion channel 1; VEGF, vascular endothelial growth factor.

Recent studies have linked autophagy dysfunction to NAD depletion. In autophagy-deficient cells, impaired mitophagy causes mitochondrial dysfunction and increased ROS production, leading to oxidative stress and DNA damage. This results in hyperactivation of PARPs and SIRTs, which in turn depletes cellular NAD pools. NAD depletion ultimately contributes to mitochondrial membrane depolarisation due to impaired mitochondrial oxidative phosphorylation, facilitating apoptotic cell death (Kataura et al., 2022; Sun et al., 2023). Loss of NPC1, which is associated with autophagy/mitophagy dysfunction, recapitulates NAD depletion wherein pharmacological autophagy/mitophagy and NAD enhancers rescue autophagy and NAD deficits and improve cell viability in cellular models including NPC1 patient iPSC-derived neurons (Kataura et al., 2022; Kataura et al., 2024). This evidence suggests that disruption in the autophagy–NAD axis contributes to the neurodegenerative pathology of NPC1 disease (Figure 2).

## STING-mediated neuroinflammation in NPC1 disease

Emerging evidence has implicated a role of autophagy dysfunction in the innate immune response and neuroinflammation mediated by stimulator of interferon genes (STING) signaling in NPC1 disease. The cyclic GMP-AMP synthase (cGAS)–STING pathway is activated when cytosolic DNA derived from pathogens or from leakage of mitochondrial or nuclear DNA is detected by cGAS, followed by the production of [cyclic guanosine monophosphate–adenosine monophosphate (cGAMP)], which in turn activates STING. STING subsequently induces the expression of type I interferons and pro-inflammatory cytokines via interferon regulatory factor 3 (IRF3) and nuclear factor kappa-light-chain-enhancer of activated B cells (NF-kB) through activation of TANK-binding kinase 1 (TBK1) (Zhang and Zhang, 2025). In various mouse models of LSDs including NPC1 disease, it has been suggested that the accumulation of cytosolic double-stranded DNA is a common pathological feature, thought to be primarily derived from mitochondria as a consequence of impaired autophagy and mitophagy (Wang et al., 2024). This cytosolic DNA activates the cGAS–STING pathway and triggers inflammatory responses in neurons, contributing to neurodegeneration.

In contrast, another study reported that STING activation in NPC1 disease occurs independently of cGAS (Chu et al., 2021). Mechanistically, reduced cholesterol levels in the ER due to NPC1 deficiency activates sterol Regulatory Element-Binding Protein 2 (SREBP2) – SREBP cleavage-activating protein (SCAP) complex, which promotes the transcription of cholesterol biosynthesis genes. This complex interacts with STING and facilitates its transport to the Golgi, a step required for STING activation, thereby shifting STING into a primed state. At the same time, STING expression levels, which are regulated by lysosomal degradation, are elevated in NPC1-deficient cells due to lysosomal dysfunction. This results in boosting of STING-mediated inflammatory signaling, and induces neuroinflammation primarily involving Purkinje cells and microglia, and ultimately neurodegeneration (Chu et al., 2021). Although these studies have not reached consensus regarding the requirement for cGAS or the principal cell types responsible for neuroinflammation, both suggest that STING-mediated inflammatory signaling contributes to the pathogenesis of NPC1 disease.

## Rare to common conditions: shared mechanisms with major neurodegenerative diseases

Many of the cellular deficits observed in NPC1 disease are also present in major neurodegenerative disorders such as Alzheimer’s disease (AD), Parkinson’s disease (PD), and amyotrophic lateral sclerosis (ALS), suggesting that these alterations may represent common pathogenic mechanisms underlying neurodegeneration. First, variants in numerous lysosome-related genes, including glucosylceramidase beta 1 (GBA1), leucine rich repeat kinase 2 (LRRK2), and C9orf72, are risk factors for PD, frontotemporal dementia (FTD), and ALS (Udayar et al., 2022). In Alzheimer’s disease, genetic risk factors such as APOE and PICALM are also linked to dysfunction of the autophagy–lysosome system (Van Acker et al., 2019). Moreover, perturbations in autolysosome formation and maturation have been shown to accelerate amyloid-β deposition in several AD models (Lee et al., 2022). Hyperactivation of mTORC1 observed in NPC1 disease has likewise been reported in AD, PD, and ALS/FTD, which can suppress autophagy initiation (Shao et al., 2020; Querfurth and Lee, 2021; Khan et al., 2023). These findings suggest that autophagy impairment in neurodegenerative diseases occurs at multiple levels: inhibition of autophagy initiation through mTORC1 hyperactivation at early stage, and defects in autophagosome–lysosome fusion and improper degradation of autophagic cargo within lysosomes at the final stage of the pathway. Consequently, neurodegenerative disease-associated aggregate-prone proteins fail to be efficiently cleared, ultimately leading to protein aggregation and fibril/plaque formation—the pathological hallmarks of many neurodegenerative disorders.

Mitochondrial dysfunction has long been implicated in neurodegenerative diseases (Suomalainen and Nunnari, 2024). In PD, mutations in genes directly involved in mitochondrial maintenance, such as PARK7/DJ-1, as well as in key mitophagy regulators PTEN induced kinase 1 (PINK1) and parkin RBR E3 ubiquitin protein ligase (PRKN), lead to impaired mitochondrial quality control and contribute to disease development (Henrich et al., 2023). Mitochondrial dysfunction has also been reported to occur at early stages in AD and ALS (Venkataraman et al., 2022; Schweingruber et al., 2025). Moreover, declining levels of NAD and neuroprotective effects of NAD boosting have also been consistently observed across neurodegenerative disease models (Lautrup et al., 2019; Dolle and Tzoulis, 2025; Kocak et al., 2025). In PD patients, oral administration of the NAD precursor nicotinamide riboside (NR) has shown modest clinical improvement, while promising benefits have been reported in mouse models of AD and ALS (Harlan et al., 2020; Brakedal et al., 2022; Ai et al., 2025). Furthermore, increasing evidence from mouse models and iPSC-based studies indicates that inflammation mediated by the STING pathway contributes to neurodegeneration in AD, PD, and ALS (Hinkle et al., 2022; Carling et al., 2024; Marques et al., 2024). Notably, in TAR DNA-binding protein 43 (TDP-43)-associated ALS models, activation of the cGAS–STING pathway triggered by mitochondrial DNA release has been shown to contribute to disease pathogenesis, highlighting a mechanistic similarity with NPC1 disease (Yu et al., 2020; Wang et al., 2024).

The greatest risk factor for neurodegenerative diseases is aging. The cellular deficits, namely impaired autophagy/mitophagy, mitochondrial dysfunction, NAD decline, and chronic inflammation involving the cGAS–STING pathway, are all recognized hallmarks of aging (Gulen et al., 2023; Lopez-Otin et al., 2023; Jimenez-Loygorri et al., 2024; Kelly et al., 2024). In sporadic neurodegenerative diseases, environmental factors and aging, may induce these intracellular deficits either sequentially or simultaneously, and their integrated and synergistic effects may ultimately trigger neuronal degeneration.

## Discussion

### Molecular mechanisms linking autophagy dysfunction to neurodegeneration in NPC1 disease

Neurodegeneration in NPC1 disease can arise from multilayered disruption of cellular homeostasis initiated by lysosomal lipid accumulation. NPC1 deficiency impairs autophagy, thereby disrupting autophagic flux and preventing the clearance of damaged intracellular compartments (Sarkar et al., 2013; Lee et al., 2014; Maetzel et al., 2014). In particular, defective mitophagy results in the accumulation of dysfunctional mitochondria, leading to loss of mitochondrial membrane potential and release of cytochrome c, which triggers caspase-dependent apoptosis (Onishi et al., 2021; Kataura et al., 2022). Simultaneously, increased mitochondrial ROS and oxidative stress activate NAD^+^-dependent enzymes such as PARPs and SIRTs, resulting in depletion of the cellular NAD pool (Kataura et al., 2022; Sun et al., 2023). NAD deficiency compromises mitochondrial membrane potential maintenance and contributes to triggering apoptotic cell death (Wilson et al., 2023; Yusri et al., 2025). Importantly, neuronal death mediated by NAD depletion has also been observed in iPSC-derived neurons from NPC1 patients, supporting that the disruption of the autophagy–NAD axis underlies in NPC1 pathogenesis (Kataura et al., 2022; Kataura et al., 2024). Additionally, leakage of mitochondrial DNA into the cytosol due to impaired mitophagy activates the cGAS–STING pathway (Wang et al., 2024). At the same time, NPC1 deficiency-mediated abnormal cholesterol distribution promotes STING priming and prevents its lysosomal degradation, thereby boosting STING signaling (Chu et al., 2021). As a result, neuroinflammation can be chronically activated and contribute to neurodegeneration in NPC1 disease.

On the other hand, other types of cell death, including necroptosis, pyroptosis and ferroptosis, have also attracted attention as a potential cell death mechanism in NPC1 disease. Necroptosis is a programmed necrotic cell death pathway mediated by the receptor-interacting protein kinase 1 (RIPK1)– receptor-interacting protein kinase 3 (RIPK3)– mixed-lineage kinase domain-like protein (MLKL) complex, and the activated MLKL translocates to the plasma membrane and causes membrane rupture (Cougnoux et al., 2016). Importantly, necroptosis can be triggered by inflammatory signals; therefore, chronic elevation of pro-inflammatory cytokines such as tumor necrosis factor-alpha (TNFα) and interleukin-6 (IL-6) induced by STING signaling may promote necroptotic cell death (Murthy et al., 2020). Since necroptosis results in the release of intracellular contents, it can further activate immune responses in surrounding cells, thereby amplifying neuroinflammation and worsening disease pathology (Cougnoux et al., 2018). Alongside, STING can promote activation of the NLR family pyrin domain containing 3 (NLRP3) inflammasome, leading to caspase 1-mediated IL-1β activation and pyroptosis (Zhang C. et al., 2025). Furthermore, increased ROS and oxidative stress caused by mitochondrial dysfunction may promote lipid peroxidation and increase susceptibility to ferroptosis (Sanchez et al., 2025).

As summarized in this review, major neurodegenerative diseases share a range of molecular and cellular alterations with NPC1 disease, including impairment of the autophagy–lysosome pathway, mitochondrial dysfunction, NAD deficit, and chronic inflammation mediated by the cGAS–STING pathway, all of which are associated with aging. Lessons from the molecular pathogenesis of NPC1 disease suggest that these intracellular deficits may not act independently. Rather, they may form interconnected feed-forward loops that collectively drive neurodegeneration.

### Clinical implications for NPC1 disease and major neurodegenerative disorders

Based on the above-mentioned interwoven pathological mechanisms, potential therapeutic interventions can target the following key nodes: (1) restoring autophagy-lysosomal function; (2) improving mitochondrial quality and energy metabolism; (3) inhibiting chronic neuroinflammation. In 2024, arimoclomol was approved by the FDA as a new therapeutic agent for NPC1 disease. Arimoclomol is thought to improve proteostasis by upregulating the molecular chaperone HSP70, thereby enhancing cellular survival particularly under lysosomal stress conditions (Mengel et al., 2021). Interestingly, arimoclomol has also been shown to ameliorate protein aggregation pathology and suppress inflammation in models of AD and PD (Xavier-de-Britto et al., 2025).

Targeting the autophagy–NAD axis has shown cytoprotective effects not only in various neurodegenerative conditions but also in NPC1 disease. A number of FDA-approved, mTORC1-independent autophagy inducers have shown protective effects in cell models of NPC1 disease (Maetzel et al., 2014). One of these autophagy-inducing drugs is carbamazepine, which has been shown to ameliorate disease-relevant phenotypes in animal models of AD, FTD and ALS (Panda et al., 2019). NAD boosting strategies have also demonstrated neuroprotective effects against neurodegeneration in NPC1 disease (Kataura et al., 2022; Kataura et al., 2024). Likewise, a number of studies have reported protective effects of NAD augmentation in major neurodegenerative diseases, and clinical translation is currently underway (Wilson et al., 2023; Dolle and Tzoulis, 2025; Kocak et al., 2025). Given the potential benefits of autophagy–NAD axis and the challenges associated with central nervous system drug delivery strategies (Nance et al., 2022; Kocak et al., 2025), future studies could explore the convergent or sequential effects of blood–brain-barrier penetrant autophagy and NAD enhancers in animal and patient-derived brain organoid models.

Inhibition of STING signaling can also be a therapeutic approach in NPC1 disease although no clinical drugs are currently available. In NPC1 disease models, the STING inhibitor C-176 successfully suppresses inflammatory cytokine expressions (Chu et al., 2021). Furthermore, it has been reported that lithium improves neurodegenerative phenotypes in *Npc1* mutant mouse via impeding STIGN/SREBP2 activation (Han et al., 2023). Mitigation of neuroinflammation by the STING inhibitor H-151 has also been reported in models of AD and PD (Hinkle et al., 2022; Xie et al., 2023; Chung et al., 2024).

Taken together, pharmacological strategies that appropriately correct dysfunction of the autophagy/mitophagy system and related cellular defects—including mitochondrial dysfunction, NAD depletion, and neuroinflammation—in the integrative manner may function not only as therapeutic approaches for NPC1 disease but also as disease-modifying strategies applicable across multiple neurodegenerative disorders.
